# Altered DNA methylation of the *ABO* gene is associated with differential plasma levels of von willebrand factor and E‐selectin

**DOI:** 10.1111/trf.18342

**Published:** 2025-08-22

**Authors:** Tianai Lou, Jamie A. Sugrue, Etienne Patin, the Milieu Intérieur Consortium, Laurent Abel, Andres Alcover, Hugues Aschard, Philippe Bousso, Nollaig Bourke, Petter Brodin, Pierre Bruhns, Nadine Cerf‐Bensussan, Ana Cumano, Christophe D'Enfert, Ludovic Deriano, Marie‐Agnès Dillies, James Di Santo, Gérard Eberl, Jost Enninga, Jacques Fellay, Ivo Gomperts‐Boneca, Milena Hasan, Gunilla Karlsson Hedestam, Serge Hercberg, Molly A Ingersoll, Olivier Lantz, Rose Anne Kenny, Mickaël Ménager, Frédérique Michel, Hugo Mouquet, Cliona O'Farrelly, Etienne Patin, Antonio Rausell, Frédéric Rieux‐Laucat, Lars Rogge, Magnus Fontes, Anavaj Sakuntabhai, Olivier Schwartz, Benno Schwikowski, Spencer Shorte, Frédéric Tangy, Antoine Toubert, Mathilde Touvier, Marie‐Noëlle Ungeheuer, Christophe Zimmer, Matthew L. Albert, Darragh Duffy, Lluis Quintana‐Murci, Lluis Quintana‐Murci, Darragh Duffy, Cliona O'Farrelly

**Affiliations:** ^1^ School of Medicine Trinity College Dublin Dublin Ireland; ^2^ Translational Immunology Unit Institut Pasteur Paris France; ^3^ Human Evolutionary Genetics Unit Institut Pasteur, Université Paris Cité Paris France; ^4^ Chair Human Genomics and Evolution Collège de France Paris France; ^5^ School of Biochemistry and Immunology Trinity College Dublin Dublin Ireland

## Abstract

**Background:**

The ABO blood group system is associated with differential susceptibility to thrombotic vascular diseases. ABO is also known to be a strong *trans*‐protein quantitative trait locus for plasma proteins involved in cell adhesion and hemostasis.

**Study Design and Method:**

To further investigate these associations, we integrated epigenomic, genomic, and proteomic data from the *Milieu Intérieur* cohort. We used the rs8176719 SNP to classify donors as either type O or non‐O, and used linear models to compare levels of 229 plasma proteins in 400 donors, including age, sex, cytomegalovirus serostatus, and secretor status as covariates.

**Results:**

We observed increased levels of soluble E‐selectin and decreased levels of von Willebrand Factor (vWF) in O donors compared with non‐O donors. By performing an epigenome‐wide association study, we identified 23 differentially methylated CpG sites between blood types, which were all located in the *ABO* gene. Notably, CpG sites in the *ABO* promoter region of type O donors were less methylated than those of the non‐O donors. Using mediation analysis, we found that these differences in DNA methylation partially explained the effects of blood group on differential E‐selectin and vWF plasma levels.

**Discussion:**

We find differentially methylated CpG sites between blood types and provide new evidence that ABO blood group status affects circulating levels of specific proteins.

**Abbreviations**
ACE: angiotensin converting enzyme
CMV: cytomegalovirus
CNS: central nervous system
CpG: 5'‐C‐phosphate‐G‐3'
CVD: cardiovascular disease
EWAS: epigenome wide association study
FDR: false discovery rate
FVIII: factor VIII
Gal: galactose
GalNAc: N‐acetylgalactosamine
GWAS: genome wide association studies
ISBT: International Society of Blood Transfusion
MI: Milieu Interieur
NCBI: National Center for Biotechnology Information
PECAM: platelet endothelial cell adhesion molecule
pQTL: protein quantitative trait locus
RBCs: red blood cells
sE‐selectin: soluble E‐selectin
SNP: single nucleotide polymorphism
TGF‐beta: transforming growth factor beta
TIMP: tissue inhibitor of metalloproteinases
TSS: transcription start site
UCSC: University of California Santa Cruz
VEGFR: vascular endothelial growth factor receptor
VTE: venous thromboembolism
vWF: von Willebrand Factor

## 1 INTRODUCTION

The ABO blood group system is a critical determinant of compatibility in transfusion medicine and is also associated with differential susceptibility to thromboembolic and cardiovascular disease.^1^, ^2^, ^3^, ^4^ The presence or absence of specific modifications to the H antigen on the surfaces of erythrocytes determines whether an individual is type O or non‐O. The H antigen of type O individuals remains unmodified. In contrast, in non‐O individuals, the H antigen is extended with either *N*‐acetylgalactosamine (GalNAc) or galactose (Gal) to form the A or B antigen, respectively. Non‐O individuals are categorized into A, B, or AB blood groups. This extension is determined by the specificity of the glycosyltransferase encoded in the *ABO* gene. The ABO blood group system is complex, with over 200 alleles primarily involving exonic variants, as cataloged by the International Society of Blood Transfusion (ISBT).^5^ Genetic variation at the *ABO* gene can be divided into three main groups: the *O* alleles, which predominantly have a truncated enzyme due to a single nucleotide deletion at codon 261 (c.261delG) and lack transferase activity. It is also important to acknowledge that while c.261delG is the most common mutation resulting in type O blood, approximately 2% of all O alleles do not have the c.261delG mutation and instead have the c.802G>A mutation, which encodes a full‐length but inactive glycosyltransferase.^6^ The *A* and *B* alleles encode functional A and B glycosyltransferases, which differ in sequence by only four amino acids, based on four single nucleotide substitutions at codons 176, 235, 266, and 268 in the *ABO* gene.^7^, ^8^ ABO(H) antigens are primarily expressed on red blood cells (RBCs) but have also been found on epithelial cells, platelets, endothelial cells, and to a lesser extent on lymphocytes.^9^, ^10^, ^11^, ^12^ In addition, ABO(H) glycans have been identified on several serum proteins such as von Willebrand Factor (vWF) and factor VIII (FVIII).^13^ As vWF interacts with coagulation FVIII, it may influence the stability of FVIII. Alterations in FVIII glycosylation could also modify its half‐life or affect its interactions with the immune system, suggesting that blood group antigens may be important in systemic diseases beyond transfusion reactions.^14^, ^15^

Several studies have shown that type O individuals have a reduced risk for thrombotic vascular disease, and a recent meta‐analysis including 21,010 thrombotic events showed that type O individuals have lower odds ratios for venous thromboembolism (VTE), cerebral ischemic events, and myocardial infarction.^1^, ^2^, ^3^, ^4^ Furthermore, several genome‐wide association studies (GWAS) identified the *ABO* locus as one of the genome‐wide significant loci associated with VTE and cardioembolic stroke.^16^, ^17^, ^18^, ^19^ One potential mechanism underlying the association between ABO blood group and risk of thrombotic vascular diseases is based on the long‐recognized link between ABO and plasma protein levels. The *ABO* locus is a strong *trans*‐protein quantitative trait locus (pQTL) for plasma proteins involved in cell adhesion and hemostasis, with type O individuals having approximately 19% higher levels of plasma E‐selectin and 30% lower levels of plasma vWF compared to non‐O individuals.^20^, ^21^, ^22^ Notably, the *ABO* gene has been shown to have a recessive effect on plasma protein levels.^21^ It is thought that lower levels of serum vWF in type O individuals may have a protective role against pathologic clot formation.^23^, ^24^

Previous studies have shown that covalently linked ABO blood group determinants can regulate the susceptibility of plasma protein proteolysis.^25^, ^26^ However, how ABO blood type contributes to variability in plasma protein levels remains largely unknown. Variation in methylation could influence biological processes by regulating gene expression, either as a cause or as an outcome in response to physiological conditions or disease contexts.^27^ Changes in DNA methylation have been associated with cardiovascular disease and type 2 diabetes.^28^, ^29^ Therefore, multi‐omics analysis of well powered and characterized healthy human cohorts may provide new perspectives on the effects of ABO blood group on plasma proteins and subsequent disease susceptibility.

The *Milieu Interieur* (MI) study consists of a cohort of 1000 unrelated, healthy individuals of Western European descent, evenly stratified by sex (500 males and 500 females) and age across five decades of life (20–69, *n* = 200 per decade). The aim of the MI project is to better understand genetic and environmental determinants of immune variability.^30^ Previous studies from MI have shown that age, sex, genetics, cytomegalovirus (CMV) serostatus, and immune cell counts are crucial drivers of variation in human immune responses.^31^, ^32^ More specifically, blood protein differences in response to influenza stimulation were shown to be associated with the RH system in males but not females, and a genetic analysis of plasma protein levels confirmed the *ABO* locus as a *trans*‐pQTL.^33^, ^34^ We hypothesized that an in‐depth exploration of ABO blood group associations with plasma proteins may provide new understanding of the biological mechanisms linking these different factors.

## 2 STUDY DESIGN AND METHODS

### 2.1 Study population

One thousand unrelated healthy individuals were included in the MI study, evenly stratified by sex (500 males and 500 females) and across five decades of life (20–69, with 200 individuals per decade). Only individuals of western European descent (i.e., French citizens for whom the last three generations of ancestors were from mainland France) were included.^30^ The MI study was approved by the Comité de Protection des Personnes—Ouest 6 (Committee for the protection of persons) on June 13, 2012, and by French Agence nationale de sécurité du médicament (ANSM) on June 22, 2012. The study was sponsored by Institut Pasteur (Pasteur ID‐RCB Number: 2012‐A00238‐35) and was conducted as a single center interventional study without an investigational product (NCT01699893). The study was designed and conducted in accordance with the Declaration of Helsinki and good clinical practice, with all subjects giving informed consent. The samples and data used in this study were formally established as the MI biocollection (NCT03905993), with approvals by the Comité de Protection des Personnes—Sud Méditerranée and the Commission nationale de l'informatique et des libertés on April 11, 2018.

### 2.2 Genotype‐based imputation of the ABO blood group status

All participants (1000 individuals) were genotyped using two genome‐wide SNP arrays, HumanOmniExpress and HumanExome BeadChips.^31^ SNP data was processed and filtered as previously described.^31^ The ABO blood group status (type O or type non‐O) of 998 individuals were determined based upon their genotype for the rs8176719 polymorphism: G/G, G/−, and −/− individuals were assigned the non‐O, non‐O, and O status, respectively. In total, 958 donors were retained for an epigenome‐wide association study (EWAS), and 400 specifically for plasma protein analysis, based on completeness of available datasets. For males, *n* = 209 type O and *n* = 289 type non‐O. For females, *n* = 196 type O and *n* = 304 type non‐O. The non‐O group was further determined as blood types A, B, or AB based on genotypes at two other SNPs, rs8176746 (Leu266Met) and rs8176747 (Gly268Ala), as previously described.^5^ Briefly, blood type A was defined by the following genotype combinations: rs8176719 = −/G, rs8176746 = G/G, and rs8176747 = C/C; or rs8176719 = G/G, rs8176746 = G/G, and rs8176747 = C/C. Blood type B was defined by two possible genotype combinations: rs8176719 = −/G, rs8176746 = G/T; or rs8176719 = G/G, rs8176746 = T/T. Blood type AB was defined by the genotype rs8176719 = G/G, rs8176746 = G/T, and rs8176747 = C/G. Finally, blood type O was defined by the genotype rs8176719 = −/−.

**Table:** 

rs8176719	rs8176746	rs8176747	Type O/non‐O status	ABO blood groups
−/−			Type O (*n* = 405)	O/O (*n* = 405)
−/G	G/G	C/C	Non‐O (*n* = 593)	A/O (*n* = 366)
G/G	G/G	C/C	Non‐O	A/A (*n* = 86)
−/G	G/T	C/G	Non‐O	B/O (*n* = 79)
G/G	T/T	G/G	Non‐O	B/B (*n* = 10)
G/G	G/T	C/G	Non‐O	A/B (*n* = 52)

### 2.3 Secretor and non‐secretor status of the MI cohort

Secretor status refers to the ability to secrete ABO blood group antigens into bodily fluids and is determined by the *FUT2* gene.^35^ It influences plasma protein levels, such as vWF,^36^ by affecting antigen expression on endothelial cells as well as protein production and clearance. Additionally, secretor status affects susceptibility to Norwalk virus and contributes to variability in plasma protein profiles, making it an important factor in regression models.^37^, ^38^ The secretor status of all donors from the MI cohort was determined based upon their genotype for the rs601338 polymorphism: G/G, G/A, and A/A individuals were assigned secretor, secretor, non‐secretor status, respectively.^35^ We included secretor status as one of the covariates in linear regression models (lm[Log_2_[plasma protein levels] ~ ABO blood type + SEX + AGE + CMV serostatus + Secretor status]) to assess differences in plasma protein levels between type O and non‐O individuals.

### 2.4 DNA methylation profiling

Genomic DNA was extracted from the blood of 958 individuals of the MI cohort and treated with sodium bisulfite (Zymo Research, California, USA). CpG methylation profiles were generated using Infinium MethylationEPIC BeadChip (Illumina, California, USA) at 866,836 CpG sites in the human genome, as described elsewhere.^39^ After noob normalization, *M*‐values were corrected for batch effects using the ComBat function, from the sva R package. *β* values were calculated by $\beta =2^{M}/\left(2^{M}+1\right)$. Here, we used *β* values for all CpG sites for downstream analyses.

### 2.5 Plasma protein levels in 400 MI donors

A subset of the MI cohort consisting of 400 donors, equally stratified by sex and two decades of life (30–39 and 60–69), was previously selected for plasma protein quantification.^34^ The concentrations of 229 plasma proteins from these 400 donors were quantified by Luminex multi‐analyte immunoassays as previously described.^34^ Proteins measured included angiogenesis markers, tissue remodeling proteins, cytokines, chemokines, metabolic markers, hormones, growth factors, acute‐phase reactants, cancer markers, kidney damage markers, and central nervous system (CNS) biomarkers. The levels of all 229 proteins were Log_2_‐transformed for downstream analyses.

### 2.6 CMV serology

CMV serostatus was assessed by a clinical‐grade IgG assay based on the manufacturer's instructions.^31^ Anti‐CMV IgG was measured on the Unicel Dxl 800 Access platform using a chemiluminescence‐based kit (CMV IgG kit, Beckman Coulter). CMV serostatus was included in our analyses as a covariate as CMV has previously been shown to have a specific effect on secretory cytokine levels by altering the number, activation status, and transcriptional profiles of blood cell populations. In particular, CMV seropositivity is associated with increased numbers of CD4^+^ memory T cells and is strongly associated with increased DNA methylation at genes related to immunosuppressive cytokine secretion, including transforming growth factor beta (TGF‐β).^39^

### 2.7 Immune cell proportions

Immune cell proportions were measured in each participant of the MI cohort (1000 individuals) using 10 eight‐color flow cytometry panels. Flow data from the stained cells were acquired using two MACSQuant analyzers, which were calibrated using MACSQuant calibration beads (Miltenyi, Germany). Flow cytometry data were analyzed using MACSQuantify software version 2.4.1229.1, as previously described.^31^

### 2.8 Statistical analysis

All data were analyzed using R Studio (version 2023.12.1 + 402, https://www.rstudio.com/). To assess differences in DNA methylation between type O and non‐O individuals, we applied EWAS using linear regression analysis with the lm() function from the R stats package, including sex, age, CMV serostatus, and 16 immune cell subset proportions as covariates (lm[CpG site ~ ABO blood type + SEX + AGE + CMV + 16 cell proportions]). *p* Values from the EWAS analysis were corrected for multiple testing using an FDR adjustment to generate *q* values. A *q* < 0.05 was considered significant.

We included sex, age, CMV serostatus, and Secretor status as covariates in linear regression models (lm[Log_2_[plasma protein levels] ~ ABO blood type + SEX + AGE + CMV + Secretor status]) to assess differences in plasma protein levels between type O and non‐O individuals. We further introduced each differentially methylated CpG site as an additional covariate in regression models (lm[Log_2_[plasma protein levels] ~ ABO blood type + SEX + AGE + CMV + Secretor status + *CpG*]). *p* Values were adjusted for false discovery using FDR correction to generate *q* values. A *q* < 0.05 was considered significant. Additionally, models were run on Log_2_ fold change of plasma protein concentrations between type O and non‐O.

Mediation analysis was performed using mediation (4.5.0) R package, with *X* = ABO blood type, *M* = CpG site, *Y* = Plasma protein. ModelXM: (lm[CpG site ~ ABO blood type + SEX + AGE + 16 cell proportions]) ModelXMY: (lm[Log_2_[Plasma protein] ~ ABO blood type + CpG site + SEX + AGE]) The R package mediation (4.5.0) was used to estimate the indirect and direct effects, employing 500 simulation nonparametric bootstrapping to generate confidence intervals (CI).

Manhattan plots were created with the R package fastman (0.1.0). Volcano plots were created using the EnhancedVolcano (1.22.0) R package. Scatterplots were created using GraphPad Prism (10.2.3). Boxplots were created using the R package ggplot2 (3.5.1). Heatmaps were generated using the R package pheatmap (1.0.12).

## 3 RESULTS

### 3.1 ABO blood status distribution in *Milieu Interieur*

The presence or absence of guanine at rs8176719, a SNP in the *ABO* gene, was used to define type O and non‐O donors in our cohort (*n* = 998). 40% of the MI donors are blood type O, while 60% are type non‐O (Figure S1A,B), and the numbers of type O or non‐O individuals were similar among males and females. The frequencies of type O and non‐O based on genotype are also comparable to those observed for Europeans in the 1000 Genomes Project data (Figure S1C).^40^ The combination of three SNPs in the *ABO* gene, rs8176719, rs8176746, and rs8176747, was used to infer the ABO blood group phenotypes, including type A, B, AB, and O. The AB phenotype was defined with G/G at the rs8176719 SNP, G/T at the rs8176746 SNP, and C/G at the rs8176747 SNP. The frequencies of these ABO blood phenotypes were also similar to those from the *Institut National de la Transfusion Sanguine* in France (Figure S1D,E).

### 3.2 Type O and non‐O blood groups affect plasma protein levels

**FIGURE 1:**
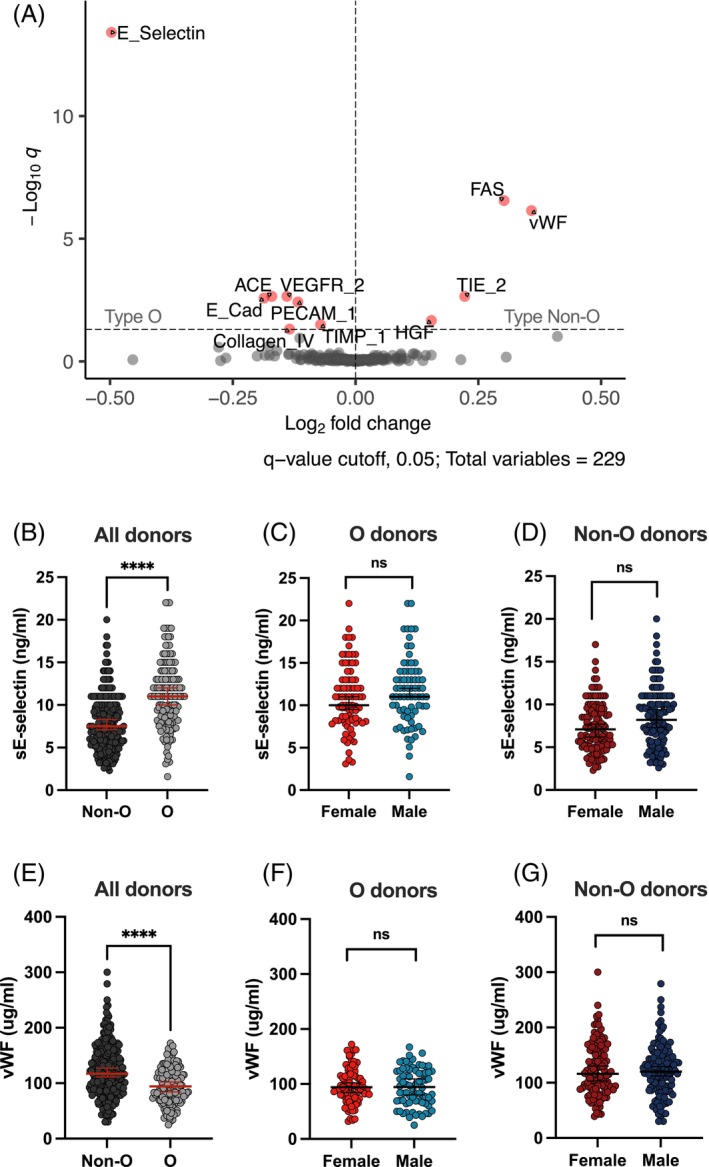
Differential plasma protein levels between type O and type non‐O individuals. Two hundred and twenty‐nine plasma proteins of 400 MI donors were quantified by Luminex multi‐analyte immunoassays. Data were Log_2_ transformed. (A) Volcano plot showing differential levels of 11 plasma proteins between type O and type non‐O individuals in MI. (B) Dot plot showing levels of soluble E‐selectin (sE‐selectin) in O individuals compared to non‐O (****, *q* < 0.0001, linear regression with FDR correction). (C and D) Dot plot showing sE‐selectin levels between females and males in MI (*q* > 0.05, FDR correction). (E) Dot plot of von Willebrand Factor (vWF) in type O and non‐O donors (****q < 0.05, linear regression with FDR correction). (F and G) Dot plot of vWF levels in plasma of females and males in MI (*q* > 0.05, FDR correction). [Color figure can be viewed at wileyonlinelibrary.com]

Two hundred and twenty‐nine protein levels in plasma from 400 healthy individuals (20–29 and 60–69 years) were quantified by Luminex multi‐analyte immunoassays. Eleven plasma protein levels were significantly different between type O and non‐O individuals (FDR *q* < 0.05) (Figures 1A and S2). Consistent with previous studies, type O individuals had higher levels of cell adhesion molecules and lower levels of serum clotting factors.^20^ Among these 11 plasma proteins, soluble E‐selectin (sE‐selectin) had the lowest *q* value and the highest absolute value of fold change (FDR, *q* = 3.88E−14; Log_2_FC = −0.4971), and vWF had the second highest absolute value of fold change (FDR, *q* = 7.16E−7; Log_2_FC = 0.358). Specifically, we observed increased levels of sE‐selectin and reduced levels of vWF in type O donors (Figure 1B,E). Given the important sex‐specific differences known to be associated with circulating proteins, we also investigated sex‐specific effects. The cohort was stratified by sex and assessed for differential plasma protein levels between type O and type non‐O males and females. Both sE‐selectin and vWF levels were similar between type O and type non‐O males and females (Figure 1C,D,F,G). Since secretor status, determined by the FUT2 gene, is known to influence plasma protein levels, including vWF, we incorporated it as a covariate in our regression models to account for its role in protein production and clearance.^37^, ^38^ Secretor status was included to better capture the variability in plasma protein profiles and ensure that differences observed between blood types were not confounded by this genetic factor.

We further stratified our cohort into the four inferred ABO blood group phenotypes (types A, B, AB, and O) and six inferred ABO blood group genotypes (types AO, AA, AB, BB, BO, and OO) by using the combination of three SNPs, rs8176719, rs8176746, and rs8176747 in the *ABO* gene (Figure S3A,B,E,F). We observed that the *ABO* locus has recessive effects on sE‐selectin levels (Figure S3B) and vWF levels (Figure S3F). We also used an additional SNP (rs1053878) to differentiate *A* ^*1*^ and *A* ^*2*^ alleles within the blood group A. Within the MI cohort, the *A* ^*1*^ allele frequency is 22.3%, while the *A* ^*2*^ allele frequency is 7.2%, which are consistent with the haplotype frequencies of *A* ^*1*^ and *A* ^*2*^ alleles in European ancestry populations reported in the meta‐analyses of the six studies across European populations.^41^ The A2 donors express four‐ to five‐fold less A antigen than A^1^ donors, and the relative amounts of H antigen are found in the following sequence of phenotypes: O > A^2^ > A^1^.^41^ We found that the levels of vWF and E‐selectin in A^2^ donors were in between the levels in type O and in A^1^ donors (Figure S3C,G). Therefore, these results suggested that the levels of E‐selectin and vWF are associated with the relative amounts of H antigen and further confirmed our findings that homozygous O individuals have significantly different levels of sE‐selectin and vWF than both heterozygous O individuals and homozygous non‐O individuals (Figure S3D,H).

### 3.3 ABO blood group affects DNA methylation levels in the *ABO* gene

**FIGURE 2:**
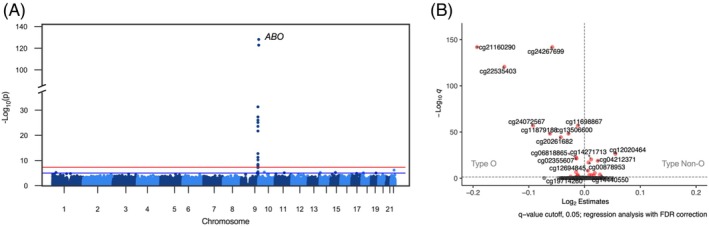
Significant epigenome‐wide associations between type O and type non‐O individuals in the *Milieu Intérieur* cohort. (A) Manhattan plot showing differentially methylated CpG sites at the ABO locus on chromosome 9 (red line, genome‐wide significance threshold *p* < 5 × 10^−8^). (B) Volcano plot of 23 CpG sites significantly associated with ABO blood status (*q* < 0.05, regression analysis with FDR correction, *n* = 958). [Color figure can be viewed at wileyonlinelibrary.com]

DNA methylation at >850,000 CpG sites in the epigenome of the 958 MI donors was previously quantified with the Illumina Infinium MethylationEPIC array.^39^ To investigate the effect of ABO blood group on variation in DNA methylation, we performed an EWAS, including sex, age, CMV serostatus, and proportions of 16 circulating immune cell subsets as covariates in the regression models. Immune cell subsets were included as we previously reported their important contribution to variability in whole blood methylation.^39^ For these 16 cell subsets, we first confirmed that they were not different (*q* > 0.05, linear regression with FDR correction) between type O and non‐O individuals. From the EWAS, we found that genome‐wide significant CpG sites associated with ABO blood type are all located in the *ABO* gene on chromosome 9 (Figure 2A). Focusing on the *ABO* gene, we identified 23 differentially methylated CpG sites between type O and non‐O individuals (*q* < 0.05, regression analysis with FDR correction, Figure 2B). Type O individuals are positively associated with 14 CpG sites and have negative associations with nine CpG sites.

### 3.4 Type O individuals are less methylated at the ABO promoter/enhancer regulatory region than non‐O individuals

**FIGURE 3:**
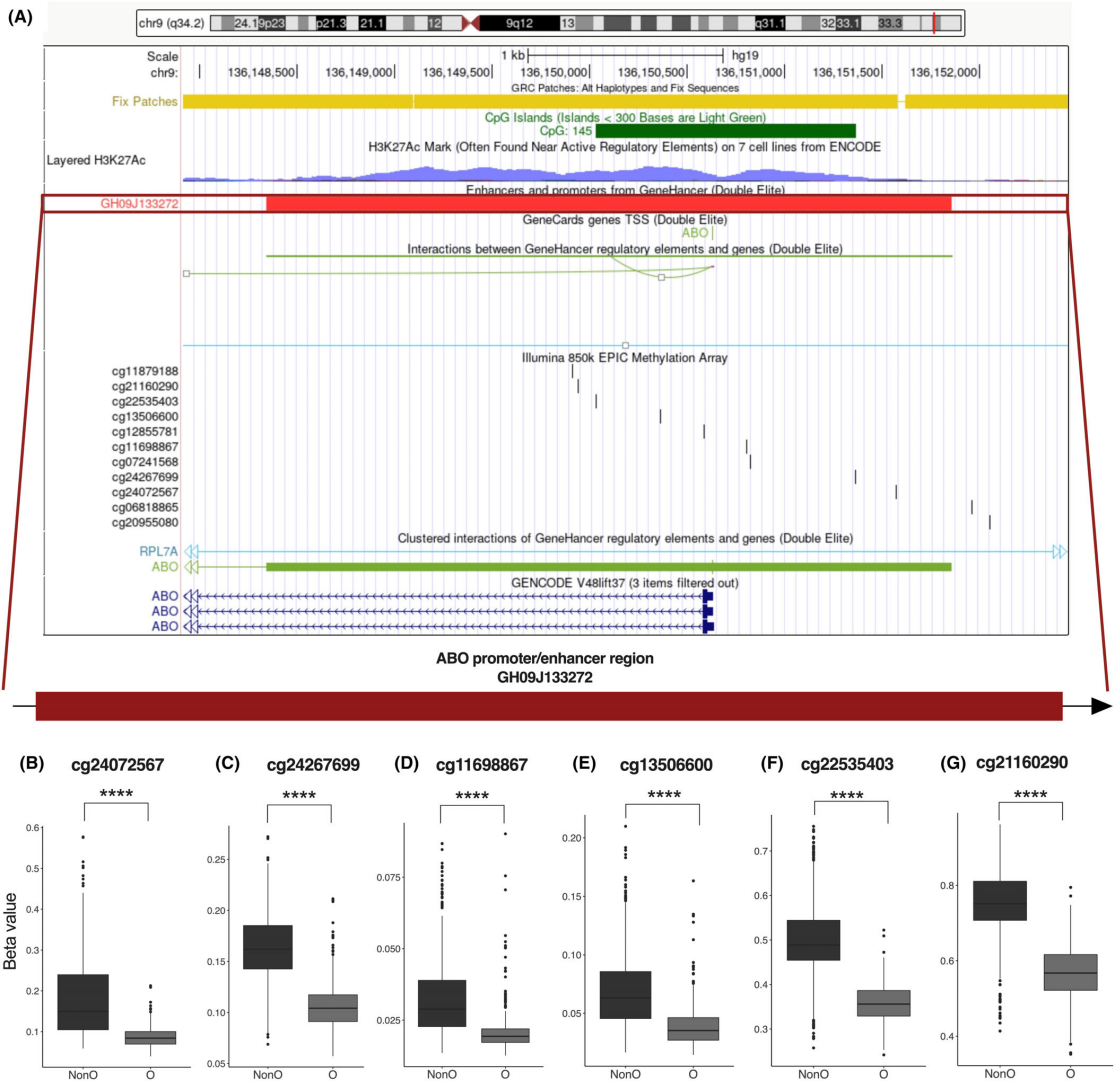
Type O donors are less methylated in the ABO promoter/enhancer region Gh09J133272 compared to non‐O donors in MI. (A) Map showing UCSC Genome Browser on Human (GRCh38/hg38) Chr9.133,272,926–133,276,404. ABO promoter/enhancer region GH09J133272. (B–G) Box plots showing beta values of methylation levels at six differentially methylated CpG sites between type O and non‐O individuals in the ABO promoter/enhancer region GH09J133272 (****, *q* < 0.0001, regression analysis with FDR correction, *n* = 958). [Color figure can be viewed at wileyonlinelibrary.com]

*ABO* gene expression is dependent upon DNA methylation at promoter and enhancer regulatory regions.^42^ By identifying the position of these 23 differentially methylated CpG sites in the *ABO* gene, we observed a novel methylation pattern at the ABO promoter/enhancer region GH09J133272 (3.5 kb, chr9: 133,272,926–133,276,404; GRCh38/hg38) between blood groups. Six differentially methylated CpG sites in the ABO promoter/enhancer regions of type O individuals were less methylated than the ABO promoter regions of type non‐O individuals (*q* < 0.05, regression analysis with FDR correction, Figure 3).

### 3.5 Changes in DNA methylation are associated with the effect of O blood type on plasma protein levels

**FIGURE 4:**
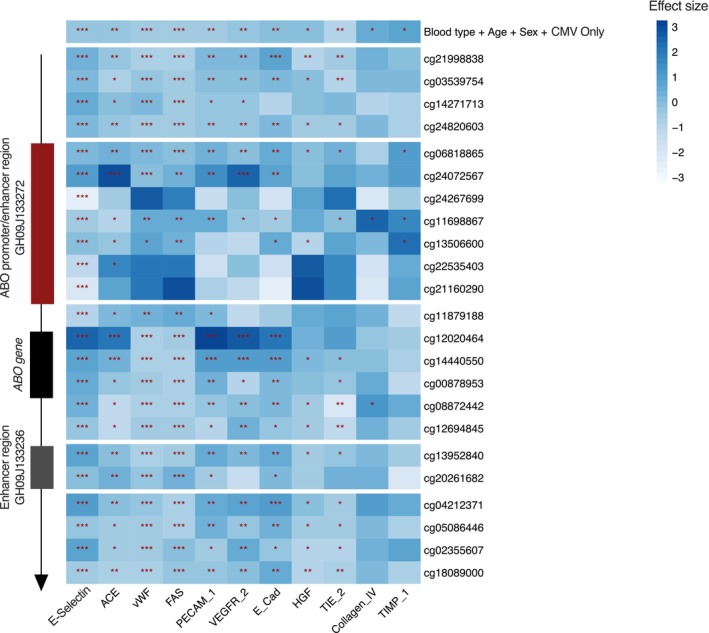
The effects of ABO blood group status on plasma protein levels are modified by DNA methylation in the MI cohort. Columns of the heatmap represent 11 differentially expressed plasma proteins by ABO blood types. Rows of the heatmap show 23 differentially methylated CpG sites between type O and type non‐O individuals in MI. The left‐most illustration represents the gene coordinates of the ABO gene, including ABO promoter/enhancer region, the ABO gene, and a downstream enhancer region. This coordinate shows the relative locations of each CpG site. Heatmap showing associations (effect size) of the ABO blood group status with plasma protein levels with either no methylation covariate (top line) or each of 23 differentially methylated CpG sites passed as a covariate in the models. *p* Value is adjusted with FDR adjustment indicated with red star (*n* = 958). CMV, cytomegalovirus. [Color figure can be viewed at wileyonlinelibrary.com]

To further investigate the possible influence of DNA methylation on differences in the plasma protein levels, we integrated the DNA methylation data with differential plasma protein levels. We individually added each of the 23 differentially methylated CpG sites in the *ABO* gene as a covariate in the regression model for each differentially expressed plasma protein (Figure 4). Since methylation levels are associated with ABO blood groups, we tested whether the ABO blood group–plasma protein associations remain when considering these differentially methylated CpG sites in our model. Figure 4 displays the distribution of effect sizes in a heatmap, which are calculated based on quantifications of the influence of ABO blood type on the plasma protein expression after adjusting for each methylated CpG site. Interestingly, we observed that CpGs located in the GH09J133272 promoter/enhancer region, when adjusting as covariates in the model, removed the association of ABO blood type with plasma proteins (Figure 4, *q* < 0.05, regression analysis with FDR correction). Therefore, DNA methylation changes are associated with the effect of ABO blood type on plasma protein levels.

### 3.6 Methylation at CpG sites of the *ABO* gene partially mediates the effect of O blood type on plasma protein levels

**FIGURE 5:**
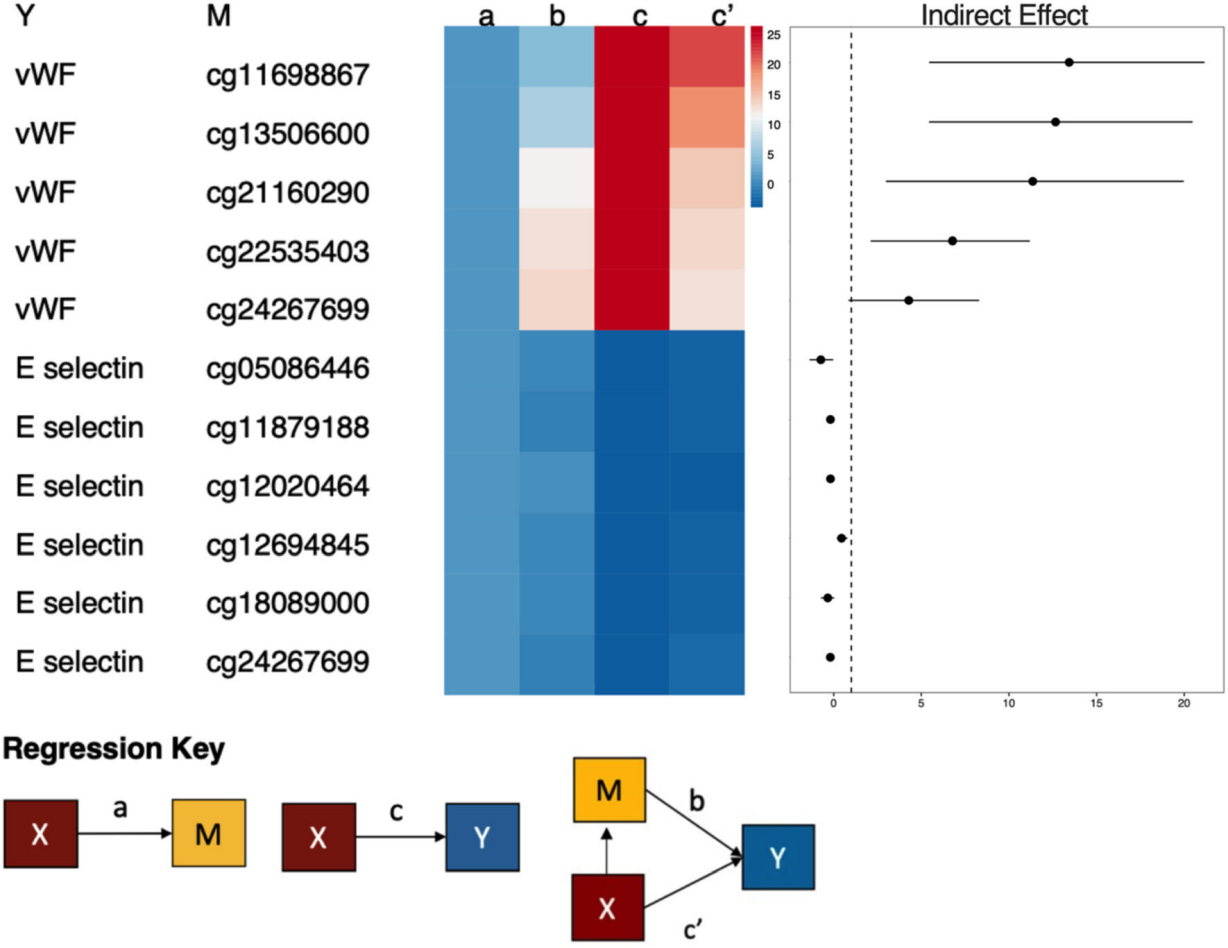
Methylation at the ABO gene partially mediates the effect of O blood type on plasma protein levels. Heatmap showing the relationship between predictor (*X*, ABO blood group status), mediating (*M*, DNA methylation levels at CpG sites), and predicted variables (*Y*, E‐selectin and von Willebrand Factor [vWF] serum levels). Only mediations with non‐zero confidence intervals for the indirect effect are shown. The right‐most box represents the estimates of the indirect effect (*c*–*c*′) bound by its lower and upper 95% confidence intervals. Paths *a*, *b*, *c*, and *c*′ are the regression coefficients for *X* ➔ *M*, *M* ➔ *Y*, *X* ➔ *Y*, and *X* + *M* ➔ *Y*, respectively. [Color figure can be viewed at wileyonlinelibrary.com]

To further interrogate the relationship between DNA methylation and plasma proteins, we performed mediation analysis, where the independent variable (*X*) is “ABO blood type,” the mediator (*M*) is “CpG site methylation” and the dependent variable (*Y*) is “plasma protein levels” (Figure 5, Regression Key). All models were adjusted for the effects of age, sex and 16 circulating immune cell types. Using this approach on two plasma protein examples (vWF and sE‐selectin), we found that methylation had a significant partial mediation effect on correlations between ABO blood status and plasma protein levels (Figure 5). The effect of ABO blood type on plasma vWF levels is mediated through methylation at five CpG sites. Methylation at six CpG sites partially mediates the relationship between ABO blood type and sE‐selectin levels.

Comparing the results from mediation analysis, we observed one shared CpG site, cg24267699, with significant partial mediation effects on both vWF and sE‐selectin. The average causal mediation effect (ACME, *c*–*c*′) was estimated at 13.231 (95% CI: 4.171–21.60, *p* value = 0.004), indicating a significant indirect effect of ABO blood group status on serum vWF levels through methylation levels at cg24267699 independent of age, sex, and 16 circulating immune cell types. Additionally, the average direct effect (ADE, *c*′) was estimated at 13.315 (95% CI: 2.544–25.03, *p* value = 0.024). The proportion of the total effect mediated by methylation was estimated at 0.498 (95% CI: 0.146–0.89, *p* value = 0.004).

## 4 DISCUSSION

In a cohort of well‐characterized healthy French individuals, we identified differential levels of 11 plasma proteins between type O and non‐O individuals. Consistent with previous studies, type O individuals had increased levels of sE‐selectin and decreased levels of vWF compared to non‐O individuals.^43^, ^44^ In line with previous studies, the ABO blood group has recessive effects on both levels of sE‐selectin and levels of vWF in the plasma,^21^, ^45^, ^46^ where homozygous O individuals (genotypes OO) are significantly different from individuals carrying either one (genotypes AO and BO) or both non‐O alleles (genotypes AA, AB, and BB).

We investigated variation in the whole blood methylome between type O and non‐O individuals from the MI cohort using EWAS and found similar methylation levels at nearly all genes between the two groups. The distinct exception was the *ABO* gene where we identified differential methylation levels at 23 CpG sites. Most previous studies of DNA methylation of the *ABO* gene have mainly focused on different individual cell types.^42^, ^47^, ^48^, ^49^ Our study quantified methylation levels using DNA samples derived from whole blood, which includes all circulating cell types. Whole blood is a complex tissue containing not only immune cells but also a significant proportion of platelets and RBCs, where the majority of H antigen expression occurs. A limitation of using whole blood for DNA methylation studies is that mature RBCs and platelets lack nuclei and therefore do not contain DNA to directly contribute to the DNA methylation signals in these analyses. The disconnect between antigen expression and DNA availability makes it challenging to interpret methylation changes, as they primarily reflect the profiles of nucleated cells, mostly white blood cells. Our previous work showed that the cellular composition of immune cells is one of the strongest predictors of DNA methylation, while intrinsic factors, such as age, sex, and CMV infection also attribute to substantial changes in DNA methylation.^39^ This highlights the importance of accounting for these factors in methylation studies of whole blood. However, we found no significant differences in the distribution of the major circulating immune cell populations between type O and non‐O individuals.

Variation in genetic DNA sequences also has an intrinsic, profound effect on DNA methylation patterns compared to external factors.^50^ We included each of 164 common SNPs from the *ABO* locus as covariates in our regression models; however, the association between DNA methylation and the ABO blood group remained significant after adjusting for these SNPs, except for the SNP rs8176719 and SNPs that are in linkage disequilibrium with rs8176719 (*D*′ ≥0.9648 and *r* ^2^ >0.8). This suggests that differentially methylated CpG sites in the *ABO* gene between blood groups are not influenced by common polymorphisms of the *ABO* locus, aside from those associated with the ABO blood group itself.

One of the most interesting findings of our study is that the promoter/enhancer region of the *ABO* gene in type O individuals is less methylated than the promoter region of non‐O individuals. Previous studies have demonstrated that DNA methylation in the promoter region plays an important role in gene silencing in a cell‐type‐specific manner.^42^, ^48^ However, with a focus on cell‐type‐specific expression, Yamamoto et al. reported a negative correlation between methylation levels at the promoter region and *ABO* gene expression. The authors suggest that cells with hypomethylated promoter regions have increased expression of ABO antigens, whereas hypermethylated promoter regions are found in cells that do not express ABO antigens. This may seem contrary to our findings that type non‐O individuals have higher levels of whole blood‐derived DNA methylation in the promoter/enhancer region, GH09J133272, of the *ABO* gene than O individuals, implying potential reduced expression of the *ABO* gene in non‐O individuals based on the repressive role of DNA methylation in gene transcription. This may also suggest that the *ABO* gene of non‐O individuals, encoding functional glycosyltransferases, requires more stringent transcriptional regulation compared to type O individuals, who encode a truncated isoform of the enzyme with no transferase activity. Distinctive enhancer structures with different transcriptional activities were identified among the allelic A, B, and O glycosyltransferase genes,^51^ which indirectly support the hypothesis of differential methylation signals between type O and non‐O individuals.

By integrating DNA methylation with differential levels of plasma proteins from ABO blood types, we found that methylation at six CpG sites within the ABO promoter region is strongly associated with the effect of ABO blood types on plasma protein levels. Specifically, when including two CpG sites, cg24267699 and cg21160290, each as a covariate in our model, the effect of the ABO blood group on the levels of 10 plasma proteins was removed. We further applied mediation analysis to assess the potential causal effects of DNA methylation on the relationship between the ABO blood status and plasma protein levels. Despite mediation analyses requiring a cautious interpretation of causality,^52^ our analysis identifies a potential role of DNA methylation in trans regulation of certain plasma protein levels. The partial mediation effects of methylation in the *ABO* gene on plasma protein (sE‐selectin, vWF) levels suggest that expression of the *ABO* gene is strongly associated with circulating protein concentrations. However, further experimental validation is needed to provide stronger direct evidence of this potential mediating effect, including, for example, kinetic analysis of gene expression and variation in DNA methylation in both the ABO glycosyltransferase and plasma proteins between type O and non‐O individuals.

Yu et al. observed differential transcriptional activity of ABO glycosyltransferase genes in gastric cancer cells but not in erythrocytes, suggesting the cell‐type‐specific enhancer effects.^51^ This may explain the associations between ABO blood groups and plasma protein levels, as these proteins are primarily synthesized in hepatocytes and immune cells. Our study, focusing on whole blood samples, reflects circulating components rather than protein synthesis sites like the liver. We therefore hypothesize that ABO expression regulation differs between erythroid and non‐erythroid lineages, with the enhancer region potentially playing a more significant role in non‐erythroid tissues involved in plasma protein synthesis. However, further studies in liver and other non‐erythroid tissues are necessary to confirm this hypothesis.

Collectively, our findings suggest a potential role of *ABO* gene expression on specific plasma protein levels. A recent study employed Mendelian randomization analysis using cis‐meQTLs as causal anchors and identified that six CpG sites at the *ABO* locus were found to have causal influence for CVD and its risk factors, including coronary heart disease (CHD), myocardial infarction as well as total cholesterol levels.^53^ One of the strongest putatively causal CpG sites in the *ABO* gene, cg24267699, was shown to be positively correlated with CHD/myocardial infarction risk factors. Our findings are supported by these studies and further suggest that significantly lower levels of DNA methylation at these six CpG sites within the ABO promoter region in type O individuals are associated with differential levels of vWF and sE‐selectin, and may further contribute to the reduced risk of CVD in these people. Our study, which identified associations between *ABO* gene methylation and differential vWF and sE‐selectin plasma levels in a healthy context, provides a new avenue for exploring the underlying mechanisms of these common diseases.

A limitation of our study is that we have simplified blood group stratification using one SNP (rs8176719) to differentiate between type O and non‐O individuals. While rs8176719 (c.261delG) is the most common mutation associated with type O, other alleles, such as c.802G>A, also contribute to the O phenotype by encoding inactive glycosyltransferases for approximately 2% of all O alleles.^5^ The frequencies of these additional O alleles could potentially impact our findings. Incorporation of multiple genetic markers in future larger scale studies would allow capture of rare variants and provide a more accurate classification of the O phenotype. In considering the broader applicability of our findings, particularly to populations beyond the Western European population reflected in the French cohort, such as East Asian or African populations, further investigation is needed. Genetic and environmental factors, including genetic drift, population migration, and regional variation in exposure to infectious diseases, may significantly influence allele frequencies and ABO blood group distributions. As a result, caution should be exercised when generalizing these results to other populations, and further studies across diverse populations are essential to validate our findings.

## AUTHOR CONTRIBUTIONS

JAS and TL analyzed the data and wrote the manuscript; EP and LQ‐M revised the manuscript; DD and COF supervised the study and edited the manuscript. All authors approved the final version of the manuscript. These authors contributed equally: DD and COF; JAS and TL.

## CONFLICT OF INTEREST STATEMENT

The authors declare no competing financial interests.

## Supporting information


**Figure S1.** The distribution of ABO blood group status in Milieu Intérieur Cohort. (A) Histogram showing distribution of the type O and type non‐O blood groups in MI as determined by the rs8176719 SNP. (B) Table showing frequencies of the type O and type non‐O blood groups in MI. (C) Pie charts from the 1000 Genome Project (from Ensembl) showing allele frequencies for rs8176719. (D) Histogram showing distribution of type A, type B, type AB, and type O individuals in MI determined by genotyping. (E) Table showing the frequencies of type A, type B, type AB, and type O individuals in MI and the Institut National de la Transfusion Sanguine in France.
**Figure S2.** Distribution of differential levels of plasma proteins between type O and type non‐O individuals in MI donors. (A–I) Dot plots showing the distribution of differential plasma protein levels by ABO blood groups (non‐O vs. O individuals) in 400 MI donors (*q* < 0.05, linear regression with FDR correction).
**Figure S3.** Distribution of sE‐selectin and vWF levels by inferred ABO blood group phenotypes and genotypes in 400 MI donors. (A) Dot plot of data showing the distribution of sE‐selectin levels by four phenotypes of the ABO blood (A, B, AB, and O). (B) Dot plot showing the distribution of sE‐selectin levels by six genotypes of the ABO blood group. (C) Dot plot showing the distribution of sE‐selectin level between A1, A2, and O alleles. (D) Dot plot showing allele dosage effect of the ABO blood groups on sE‐selectin levels (*p* < 0.05, One‐way ANOVA followed by Turkey's test, FDR correction). (E) The distribution of vWF levels by ABO blood group phenotypes. (F) Dot plot of the distribution of vWF levels by ABO blood group genotypes. (G) Dot plot showing the distribution of vWF levels between A1, A2, and O alleles. (H) Dot plot showing reduced vWF levels in homozygous O individuals compared with heterozygous O and homozygous non‐O individuals (*p* < 0.05, one‐way ANOVA followed by Tukey's test, FDR correction) non‐log transformed data are presented, mean of each group with 95% confidence interval are shown in dot plots.

**Data S2.** Differential levels of plasma proteins between type O and type non‐O individuals.

**Data S3.** Differentially methylated CpG sites between type O and type non‐O individuals.
