# Supplementary material for: Altered DNA methylation of the ABO gene is associated with differential plasma levels of von willebrand factor and E‐selectin
Source: Transfusion. 2025 Aug 22;65(9):1693–706. doi: 10.1111/trf.18342 (PMC12432810; doi:10.1111/trf.18342)

Supplemental Figure 1. The distribution of ABO blood group status in Milieu Intérieur Cohort

A.

The frequencies of type O and type Non-O in MI cohort

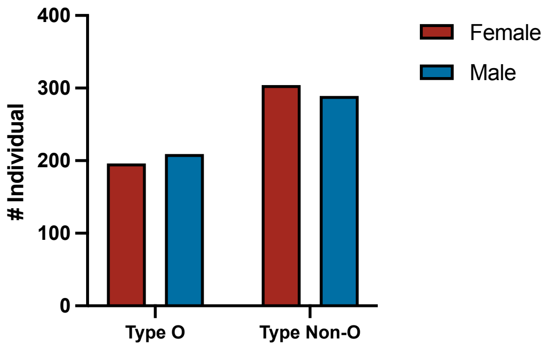

B.

The Milieu Intérieur cohort (Total : 998 individuals)			
**ABO blood type**	**Female**	**Male**	**Total**
**Type Non-O**	304	289	593 (60%)
**Type O**	196	209	405 (40%)
**Total**	500	498	998

C.

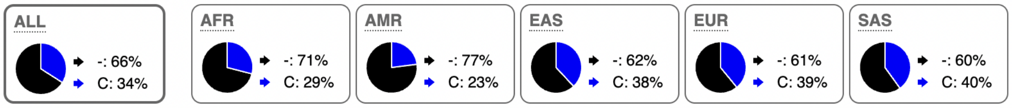

D.

The frequencies of inferred ABO phenotypes in MI cohort

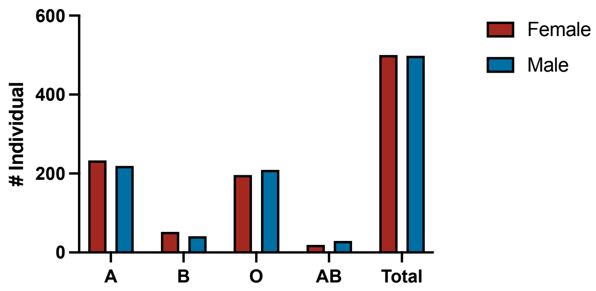

E.

		The Milieu Intérieur cohort (Total: 998 individuals)		***Institut National de la Transfusion Sanguine in France***
**ABO blood type**		**Individuals**	**Percentage**	**Percentage**
**Non-O donors**	**A**	452	45.29%	47%
	**B**	89	8.92%	9.1%
	**AB**	52	5.21%	2.9%
**Type O donors**	**O**	405	40.58%	43%

Supplemental Figure 2. Distribution of differential levels of plasma proteins between type O and type Non-O individuals in MI donors.

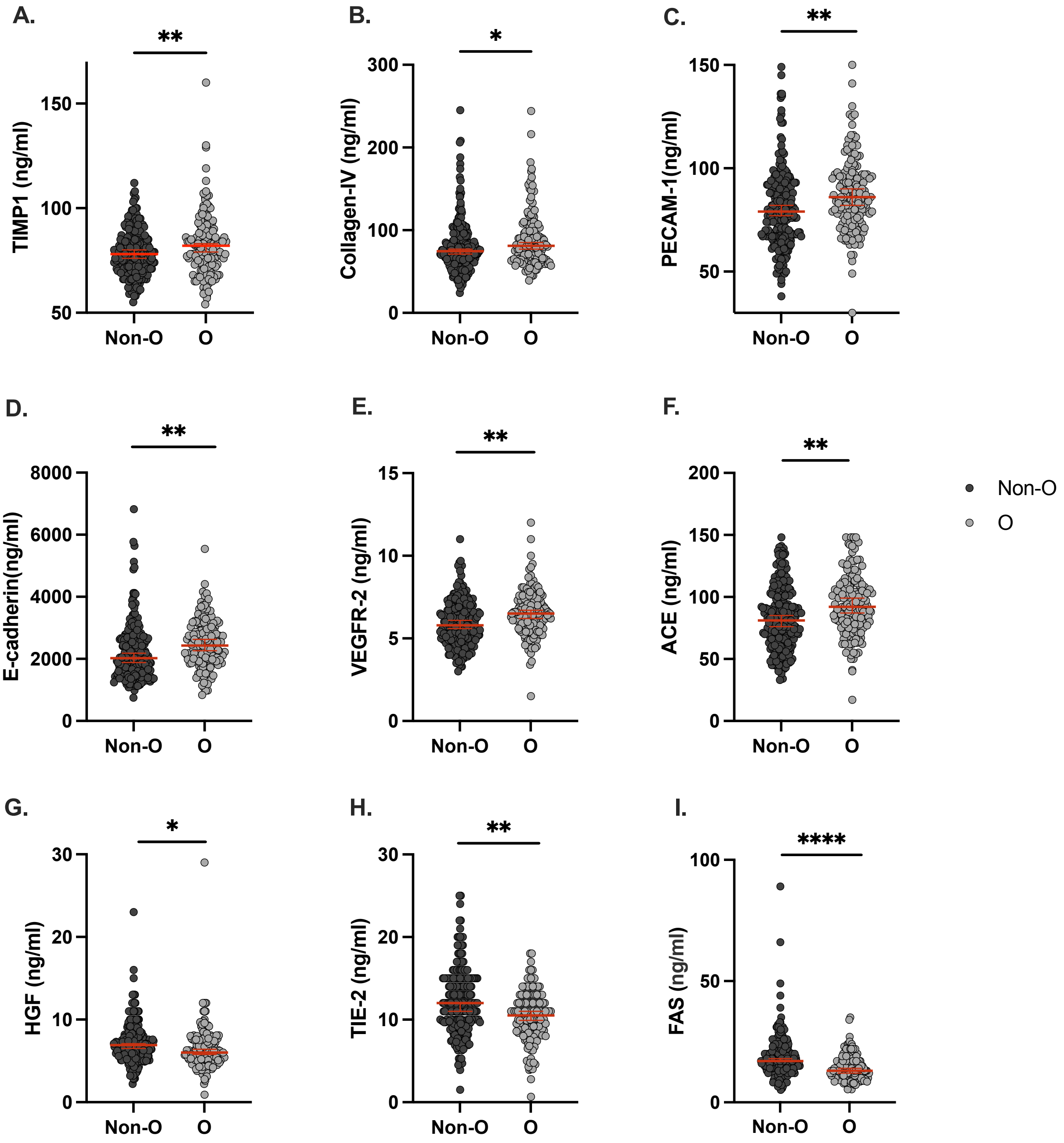

Supplemental Figure 3. Distribution of sE-selectin and vWF levels by inferred ABO blood group phenotypes and genotypes in MI donors.

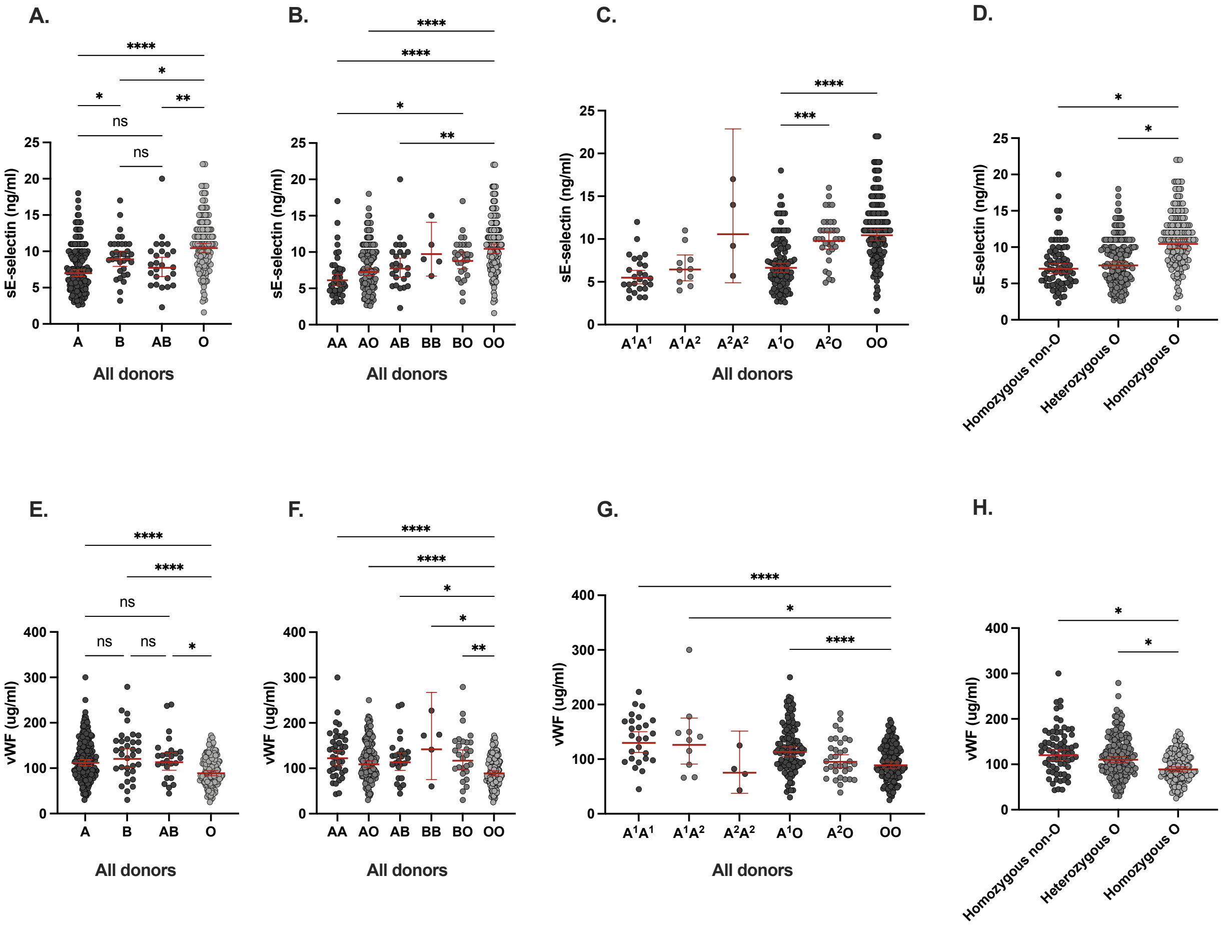

Supplement: Supplementary file 1 — Figure S1. The distribution of ABO blood group status in Milieu Intérieur Cohort. (A) Histogram showing distribution of the type O and type non‐O blood groups in MI as determined by the rs8176719 SNP. (B) Table showing frequencies of the type O and type non‐O blood groups in MI. (C) Pie charts from the 1000 Genome Project (from Ensembl) showing allele frequencies for rs8176719. (D) Histogram showing distribution of type A, type B, type AB, and type O individuals in MI determined by genotyping. (E) Table showing the frequencies of type A, type B, type AB, and type O individuals in MI and the Institut National de la Transfusion Sanguine in France. Figure S2. Distribution of differential levels of plasma proteins between type O and type non‐O individuals in MI donors. (A–I) Dot plots showing the distribution of differential plasma protein levels by ABO blood groups (non‐O vs. O individuals) in 400 MI donors (q < 0.05, linear regression with FDR correction). Figure S3. Distribution of sE‐selectin and vWF levels by inferred ABO blood group phenotypes and genotypes in 400 MI donors. (A) Dot plot of data showing the distribution of sE‐selectin levels by four phenotypes of the ABO blood (A, B, AB, and O). (B) Dot plot showing the distribution of sE‐selectin levels by six genotypes of the ABO blood group. (C) Dot plot showing the distribution of sE‐selectin level between A1, A2, and O alleles. (D) Dot plot showing allele dosage effect of the ABO blood groups on sE‐selectin levels (p < 0.05, One‐way ANOVA followed by Turkey's test, FDR correction). (E) The distribution of vWF levels by ABO blood group phenotypes. (F) Dot plot of the distribution of vWF levels by ABO blood group genotypes. (G) Dot plot showing the distribution of vWF levels between A1, A2, and O alleles. (H) Dot plot showing reduced vWF levels in homozygous O individuals compared with heterozygous O and homozygous non‐O individuals (p < 0.05, one‐way ANOVA followed by Tukey's test, FDR correction) non‐log tran [file TRF-65-1693-s001.pdf]
